# Secondary Malnutrition and Nutritional Intervention in Cholestatic Liver Diseases in Infants

**DOI:** 10.3389/fnut.2021.716613

**Published:** 2021-11-15

**Authors:** Alfredo Larrosa-Haro, Erika A. Caro-Sabido

**Affiliations:** ^1^Instituto de Nutrición Humana, Departamento de Clínicas de la Reproducción Humana, Crecimiento y Desarrollo Infantil, Centro Universitario de Ciencias de la Salud, Universidad de Guadalajara, Guadalajara, Mexico; ^2^Departamento de Salud Pública, Centro Universitario de Ciencias de la Salud, Universidad de Guadalajara, Guadalajara, Mexico

**Keywords:** malnutrition, infants, cholestatic liver disease, nutritional status, nutritional evaluation, liver transplantation

## Abstract

We aimed to conduct an updated review on the pathophysiology, diagnosis, and nutritional intervention of CCLD and secondary malnutrition in infants. Protein-energy malnutrition, impaired linear growth, fat-soluble vitamin deficiencies, and hepatic osteodystrophy can occur in up to 80% of cases. The proposed pathophysiological mechanisms include insufficient energy intake, lipid- and fat-soluble vitamin malabsorption, increased energy expenditure, altered intermediate metabolism, hormonal dysregulation, and systemic inflammation. The current approach to diagnosis is the identification of the deviation of growth parameters, body composition, and serum concentration of micronutrients, which determines the type and magnitude of malnutrition. Currently, liver transplantation is the best therapeutic alternative for the reversal of nutritional impairment. Early and effective portoenteroanatomosis can extend survival in patients with biliary atresia. Medical and dietary interventions in some storage and metabolic diseases can improve liver damage and thus the nutritional status. A proportion of patients with biliary atresia have fat-soluble vitamin deficiencies despite receiving these vitamins in a water-soluble form. With aggressive enteral nutrition, it may be possible to increase fat stores and preserve muscle mass and growth. The nutritional issues identified in the pre- and post-transplantation stages include muscle mass loss, bone demineralization, growth retardation, and obesity, which seems to correspond to the natural history of CCLD. Due to the implications for the growth and development of infants with CCLD with this complex malnutrition syndrome, innovative projects are required, such as the generation of prediction and risk models, biomarkers of growth and body composition, and effective strategies for nutritional prevention and intervention.

## Introduction

The relationship between liver physiology and human nutrition is very close, such that malnutrition is the main factor involved in the long-term clinical outcomes of chronic cholestatic liver disease (CCLD) in infants. The involvement of the liver in the different stages of the nutrition process includes intestinal digestion and absorption, transport of macro- and micronutrients to the hepatocytes where they are stored, metabolized, or transformed through complex metabolic processes, and finally, systemic distribution of these nutrients as primary nutrients or complex molecules (1). In this context, secondary malnutrition associated with CCLD can be considered as an inherent complication of advanced liver damage (in many cases, cirrhosis) rather than a condition related to external nutritional factors (2).

Chronic cholestatic liver disease occurring during the first 2 years of life comprises a heterogeneous group of nosological entities whose etiology, diagnosis, evolution, treatment, and prognosis differ, making its diagnostic and therapeutic approaches a complex challenge for clinicians (3, 4). The diseases or syndromes that share this complex pathophysiology include biliary atresia, Alagille syndrome, intrahepatic familial cholestasis, alpha-1 antitrypsin deficiency, choledochal malformation, cholestasis associated with parenteral nutrition, and metabolic diseases (2, 5–10). Early malnutrition may become a risk factor that can significantly affect growth and development (11–16). Although impairment in the nutritional status of infants with CCLD has been identified for decades and there is a large amount of evidence that explains, at least partially, its pathophysiological mechanisms, advances in the treatment of primary liver diseases, and nutritional interventions to prevent or restore nutritional deficits are partial and have low efficiency in many cases.

Therefore, our study conducted an updated review on the pathophysiology, diagnosis, and nutritional intervention of CCLD and secondary malnutrition in infants, focusing on some controversies and research gaps.

For this narrative review, we combined the published clinical experience of our group over several decades in a third-level pediatric hospital and a scoping review focused on secondary malnutrition and nutritional diagnosis and interventions in infants with CCLD. Studies published up to April 2021 were searched using six databases (Medline, EBSCO, OVID, Science Direct, JSTOR, and Wiley). The inclusion criteria were the prevalence, type, and severity of secondary malnutrition associated with CLDC and the physiopathology, diagnosis, and results of nutritional interventions.

## Pathophysiology of Secondary Malnutrition in Infants With CCLD

**Figure 1:**
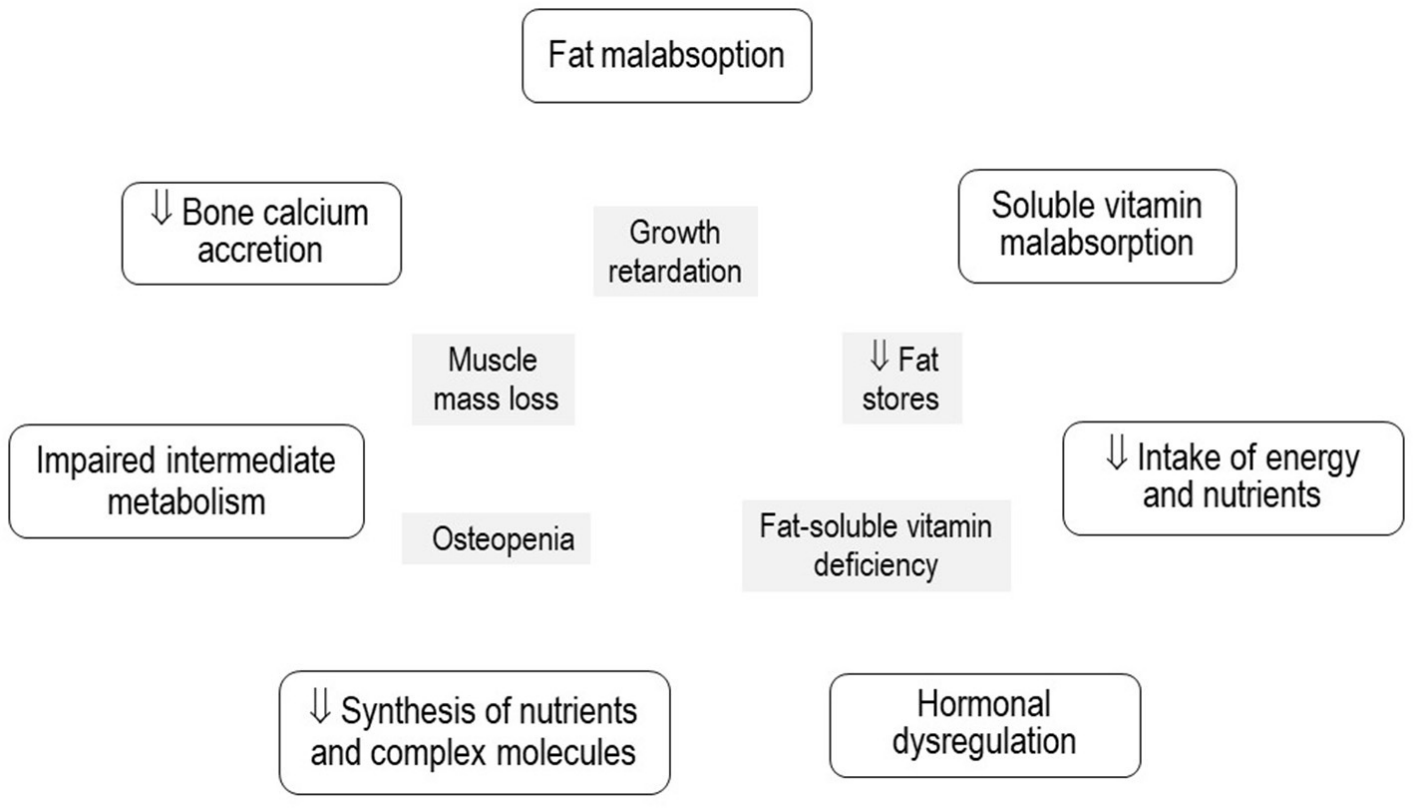
Pathophysiological mechanisms of secondary malnutrition and its expression in growth, body composition, and nutritional status of micronutrients in infants with chronic cholestatic liver diseases.

The pathophysiology of this multifaceted condition is not completely understood, it differs substantially from that of primary malnutrition, where the issue is the unavailability of food and nutrients. Once digestion and absorption have been completed after a meal, the nutrients from the diet reach the liver through the portal vein, except for fats bound to lipoproteins that arrive through the systemic circulation (1). Damage to the specialized functions of the diseased liver severely disturbs the health and homeostasis of the body, which may be particularly critical in infants during the first growth spurt (17–22) (Figure 1).

The magnitude of liver damage evaluated using liver function tests and the impairment in growth and body composition estimated by anthropometric indicators may be expressed as linear correlations that denote the close relationship between them (2, 20). The sequence of events of malnutrition in CCLD is not completely understood, as the adaptive sequence of starvation is altered owing to liver damage. The depletion of liver and muscle glycogen stores is followed by gluconeogenesis, which allows energy to be obtained from amino acids, fatty acids, and ketone bodies. Plasma amino acid extraction is reflected in the excretion of urinary N_2_ and decreased synthesis of tissue proteins, albumin, and transporter proteins (20–24). The path and sequence of the malnutrition process seem to involve consumption of fat reserves and later depletion of proteins and the muscle, demineralization of the bones, delay in linear growth, and delay in the development of the central nervous system (15, 20, 25). Other hypotheses proposed to explain the loss of muscle mass include defective ureagenesis, increased ammonia synthesis, impaired branched-chain amino acid profile, and chronic inflammation leading to increased proteolysis (26, 27).

The length and head circumference of infants with CCLD may be predicted by regression models using fat mass, fat-free mass, and bone mineral content as predictor variables (19). Osteodystrophy associated with CCLD has a multicausal etiology, including malabsorption of vitamin D, poor bone accretion of calcium, and systemic chronic inflammation with increased expression of IL-4 (28–31). In addition to decreased cell mass (31), decreased availability of energy, and impaired fat absorption (32), these factors seem to be related to the linear growth and head circumference growth retardation that is almost universally present in these patients, particularly in those with biliary atresia (2, 20, 31, 33).

Fat-soluble vitamin malabsorption associated with impaired bile salt production has been considered a significant factor for liposoluble vitamin deficiencies. In a multicenter study of 92 patients with CCLD, one-third to one-fifth had low levels of vitamins A, D, and E despite receiving supplements of fat-soluble preparations at high doses (34–37). This multiple micronutrient deficiency is associated with the complex nutritional conditions of infants with CCLD.

Although a reduction in the ingestion of infant formula and complementary feeding has been proposed as a mechanism of secondary malnutrition, this is a controversial hypothesis. Some studies have demonstrated that *PO ad libitum* intake of specialized and energy-supplemented formulas, although equal or excess of the volume ingested by a normal infant of the same age and sex are not enough to prevent or improve malnutrition (37, 38).

The gut microbiota may play a role in outcome of liver diseases through mechanisms including increased intestinal permeability, chronic systemic inflammation, production of short-chain fatty acids, and changes in metabolism (39–42). Gut microbiota and fecal transplantation could become a factor in the prevention and treatment of CCLD (42).

The probability of occurrence of protein-energy malnutrition, growth impairment, and poor bone mineralization in infants with CCLD is high (2, 18, 19), especially in settings in which there are no efficient liver transplantation programs. Factors associated with malnutrition in CCLD have been studied as independently associated variables, however, their weights in predictive models are not known. Conducting prospective studies with the development of predictive models considering other variables, such as genetic, environmental, and sociodemographic factors, and availability of diagnostic and treatment resources as efficient transplantation units, could allow the development of multivariate predictive models and thus improve the intervention strategies in different clinical settings.

## Diagnosis of Nutritional Status

Growth implies a gradual increase in the number, size, and complexity of the cells. Length is probably the best indicator of growth impairment in infants with CCLD and is directly correlated with the extent of liver damage assessed using tests, including conjugated bilirubin, albumin, or alkaline phosphatase levels (2). In patients with CCLD, it is frequent to identify a slowing in the growth rate of the skull, probably secondary to impaired growth of the central nervous system, which may have thoughtful implications in psychomotor and cognitive skills in the mid- and long terms (2, 43, 44).

The body composition evaluation of infants with CCLD focuses on fat stores, muscle mass, and bone mineral density. Various studies have shown that weight and indicators calculated based on weight and length of individuals (weight-for-age, weight-for-length, and body mass index) used worldwide for diagnostic evaluation in most liver centers (14–18, 22, 23, 34) tend to underestimate the impairment of nutritional status because patients may have ascites, distal edema, hepatomegaly, and splenomegaly (11, 20, 34, 39). This could lead to a delay in the diagnosis of acute malnutrition, which is usually the first stage in the malnutrition process, and therefore delay in nutritional interventions. A practical alternative for estimating body composition is anthropometry of the arm, a technique validated in infants with CCLD using dual X-ray absorptiometry (19). The deviation of the parameter by means of z-scores of arm measurements and indicators allows the establishment of a diagnosis of acute malnutrition and its magnitude. Its inclusion in the pediatric end-stage liver disease equation, used to estimate the relative severity of the disease and the probable survival of patients awaiting liver transplantation, could improve the diagnostic approach for candidates of liver transplantation (43, 44).

The bone mineral density in infants with CCLD is often severely affected, with z-scores well below −2 SD, especially when the serum direct bilirubin level is >2 mg/dl (30). This early and serious condition has been related to impaired absorption of vitamin D and calcium, failure of the hepatic hydroxylation phase, lack of vitamin D stores, and systemic inflammation (27–30). This bone disorder appears to be a significant predictor of linear growth retardation (19). Measuring fat-soluble vitamin A, D, and E levels in the serum of patients with CLDC is an alternative for estimating the nutritional status of these micronutrients, at least from the point of view of their serum levels (34–36).

The development of new innovative biomarkers that assess growth, body composition, and micronutrient status could be a significant advance in having a comprehensive view of this component of CCLD (45, 46).

## Nutritional Intervention

Acute and chronic malnutrition that occurs in infants with CCLD is secondary in nature. From this point of view, its therapeutic approach would be conditioned to the resolution of pathophysiological circumstances associated with primary liver disease: insufficient dietary ingestion to maintain normal growth, impaired intestinal absorption, increased metabolic rate, and impaired micro- and macronutrient metabolic pathways (47–54). Currently, the best therapeutic alternative for reversing the pathophysiology of protein-energy malnutrition, growth failure, and bone demineralization in infants with CCLD is liver transplantation (55–57). The improvement in anthropometric indicators related to body composition, such as body mass index, arm fat, and muscle areas, usually occurs 12–24 months after transplantation. However, the recovery of linear growth is minor and may not be significant (58). The assessment of body cell mass with marked potassium in children with biliary atresia after liver transplantation has shown subnormal values before transplantation that persist in the mid-term after transplantation, therefore, weight recovery is thought to be more related to an increase in fat reserves than to an increase in cell mass (31). Loss of muscle mass may be an important factor added to these severe limitations and may be responsible for the metabolic syndrome and impaired quality of life after transplantation (55, 59).

Unfortunately, therapeutic options, besides liver transplantation, aimed at modifying the natural history of cholestatic liver disease, are limited. Early and effective portoenteroanatomosis can extend survival in patients with biliary atresia, which is the most frequent and severe form of CCLD in infants (59–62). The advanced liver units and the protocols employed in the care of children with liver diseases can provide the best options to prevent the progression of liver damage and improve survival and nutritional status. The nationwide liver transplantation programs in developed countries currently offer infants with CCLD with a high short- and mid-term probability of survival with an acceptable quality of life (63, 64). However, the situation is the opposite in developing countries, where the referral of infants with CCLD to liver transplantation units is frequently delayed, and the procedure may be postponed for months, thereby increasing the risk of malnutrition (38).

Products such as ursodeoxycholic acid, anion exchange resins (such as cholestyramine), and rifampin have been used with the intention of reducing or altering the composition of the bile acid reserve, however, the clinical response to these drugs is variable and usually insufficient, and the effects on the course of the disease are limited (65, 66). Oral treatment with primary bile acids, such as cholic acid, can improve liver function in patients with bile acid synthesis defects (66). Some metabolic diseases related to enzyme deficiencies, such as galactosemia, congenital fructose intolerance, and tyrosinemia, can be managed with specific diets that allow partial or complete exclusion of the molecule or nutrients associated with liver damage. In children with type I tyrosinemia, nitisinone is an effective drug for reducing toxic metabolites in the liver and improving clinical manifestations (67). Other metabolic diseases, such as cholesterol ester storage disease and Wolman's disease, may benefit from enzyme replacement therapy. In lipid-hoarding diseases, such as Gaucher type 1 and Niemann-Pick type C, miglustat therapy can decrease lipid accumulation (66–69).

Nutritional intervention in children with CLDC under 24 months of age has been performed for several decades (45, 46). Protocols with special formulas delivered *PO* with an increase in the energy density based on cereals, glucose polymers, and fats (4, 47), the use of lipids that do not require bile acids for absorption, such as medium-chain triglycerides (4, 9, 32, 34, 36, 47), and the use of branched-chain amino acids to improve insulin resistance and favor an increase in muscle mass (70–72) are used by most liver units or pediatric gastroenterology departments. In some studies, conducted on small samples of patients with CCLD, it was shown that in infants fed these types of formula administered *PO ad libitum*, the ingested amounts were similar or slightly greater than those in healthy infants of the same age, however, this amount of energy and nutrients was not enough to achieve adequate catch-up growth. With this oral nutritional support technique, there is a high probability that patients will progressively deteriorate (37, 38, 49). The response to *PO* nutritional intervention depends on the individual situation of the liver. However, most patients with direct bilirubin levels of >2 mg/dl show a trend of decreasing growth rate and loss of fat stores and muscle mass (2).

An alternative for increasing the supply of energy and macro- and micro-nutrients is the use of enteral nutrition with the same type of formula described above, which allows the administration of a higher amount of energy and nutrients when administered with an enteral pump in a continuous infusion than when administered *PO*. The energy supply is gradually increased until it reaches 130–140% of the recommended daily intake for age with protein intake adjusted to 4–5 g/kg/day (38, 48, 49). With this technique, it is possible to increase fat stores and preserve the z-score lane of muscle mass, linear growth, and head circumference. If the enteral nutrition period should be prolonged for weeks or months, the procedure could be performed at home in 18-h infusion cycles for 6 h of rest, under the care of parents or guardians (38, 49). Enteral nutrition is indicated if patients have a realistic alternative to receive a liver transplantation in the short term.

Liver transplantation is currently the definitive treatment for most types of liver failure in both children and adults (73–76). Split grafts, live donations, immunosuppressive regimen upgrades, infectious prophylaxis protocols, and innovative surgical techniques have made it possible to reduce complications and improve survival and quality of life (55–61, 77). However, several nutritional issues become apparent once the most important medical and surgical aspects of transplantation have been controlled and exceeded. The nutritional problems that have been identified in children undergoing transplantation include muscle mass loss, diminished bone demineralization, growth retardation, and increased adiposity (31, 36, 50, 55, 56, 60, 61, 63, 72).

Loss of muscle mass occurs in non-transplanted infants with CCLD from the early stages of the disease, and its extent is related to the severity of liver damage (2, 19). As in other serious and progressive diseases, it is possible to theorize that muscle mass loss is related to a catabolic state with a negative nitrogen balance (15, 25, 49). Other reasons, such as the use of immunosuppressants, hospitalizations, infections, and renal failure, have been associated with loss of muscle mass during the pre-, intra-, and post-transplantation stages (61).

Bone mineralization disorder in infants with cholestasis accounts for a significant percentage of linear growth retardation that occurs during the first months of life (2, 19). The usual therapeutic approach to counteract this condition includes supplementation with vitamin D in its active form at high doses, guarantee of the oral intake of calcium, phosphorus, and magnesium, and exposure to sunlight, although the results of this intervention are limited (60, 61). The serum level of phosphorus, 1–25 (OH) 2D3, and total bone mineral content improved in the months following liver transplantation, probably in relation to insulin-like growth factor-I and parathyroid hormone, however, in the long term, a proportion of patients persist with osteopenia and bone metabolism disorders (61). Information on effective intervention protocols to restore bone health in addition to liver transplantation is scarce and necessary. This knowledge gap makes it necessary to develop prospective and innovative interventional studies. The possibility of enhancing growth with hormonal interventions, particularly growth hormone analogs, it would justify conducting controlled clinical trials (62).

Post-liver transplantation nutritional issues related to deficits, such as loss of muscle mass, decrease in bone mineral content, and retardation of growth, can be identified even from the clinical pre-transplantation stage. Although there are unknown factors that are involved in these clinical conditions during the intra- and post-transplantation stages, this is likely to be the same condition evolving throughout the natural history of liver disease (22, 77–83).

The prevalence of obesity in the post-transplantation stage in children is high (84–86). Children who are overweight and obese have moderate odds of remaining in this situation in the post-transplantation stage, which may be a risk factor for lower survival (86). The mechanisms underlying the increase in adiposity are not completely understood. The use of steroids during transplantation and their cumulative dose in the post-transplantation period have been considered as possible associated factors (86). Hispanic ethnicity has also been shown to be a risk factor for obesity (87).

The complexity and risks involved in CCLD should focus on the mid- or long-term outcomes in relation to growth, body composition, and neurological and psychomotor skills. Currently, the resolution of CCLD in an infant gravitates mainly around the alternative of successful liver transplantation and successful medical treatment in the long post-transplantation period. It is not known if it is possible to completely modify the complex natural history of growth and body composition impairment that occur in a high proportion of infants with CCLD. Aggressive enteral nutrition in the pre-transplantation period is an alternative, and although it does not reverse all components, it may increase fat reserves and preserve linear growth, head circumference, and muscle mass. However, the search for innovative alternatives to nutritional and medical interventions must continue, highlighting the need for developing basic and clinical research that leads to clinical trials that favor a better quality of life for patients and effective and safe interventions that provide options to modify the factors involved in acute and chronic malnutrition associated with CCLD (Table 1).

**Table 1:** Current practices, controversies, and possible future developments related to the pathophysiology, diagnosis, and nutritional intervention of CCLD associated to secondary malnutrition in infants.

**1**.	**Pathophysiology of secondary malnutrition**
	• The sequence of events of malnutrition in CCLD is not completely understood, as the adaptive sequence of starvation is altered owing to liver damage.
	• Factors associated with malnutrition in CCLD have been studied as independently associated variables; however, their involvement in predictive models is not known.
	• Recent data suggest an influence of bile on the microbiome before and after Kasai surgery.
	• Knowledge of the sequence of these pathophysiological events and their predictive value defined by predictive models could improve intervention strategies at different timing and clinical settings.
**2**.	**Diagnosis of nutritional status**
	• Weight and indicators calculated according to weight and length of individuals tend to underestimate the impairment of nutritional status because a proportion of patients have ascites, distal edema, hepatomegaly, and splenomegaly.
	• Delay in the diagnosis of acute malnutrition, which is the first stage of the malnutrition process, can postpone nutritional intervention.
	• Length z-score is an accurate indicator of growth and nutritional status. A practical alternative for estimating body composition is anthropometry of the arm, a technique validated in infants with CCLD using dual X-ray absorptiometry.
	• Measurement of fat-soluble vitamin levels in the serum and bone mineral density using dual X-ray absorptiometry complements the nutritional evaluation in a comprehensive manner.
	• Development of new biomarkers that assess growth, body composition, and micronutrient status could become a significant advance in having a comprehensive view of this component of CCLD.
**3**.	**Nutritional intervention**
	• Malnutrition in infants with CCLD is secondary in nature. Thus, its therapeutic approach would be conditioned to the resolution of the pathophysiological circumstances associated with primary liver disease.
	• Currently, the best therapeutic alternative for preventing and treating malnutrition in infants with CCLD is liver transplantation.
	• Early and successful portoenteroanatomosis can extend survival and improve growth and nutritional status in patients with biliary atresia and choledochal malformation.
	• Medical treatment to reduce or alter the composition of the bile acid reserve, control of systemic inflammation in patients with biliary atresia, diets designed for metabolic diseases related to enzyme deficiencies, and enzyme replacement therapy may contribute to the improvement of liver damage; however, their effect on the course of malnutrition is limited.
	• *PO ad libitum* ingestion of energy-fortified formulas is not enough to enhance growth and body composition in infants with conjugated bilirubin levels of >2 mg/dl. Enteral nutrition and continuous infusion can increase fat stores and preserve linear growth, muscle mass, and head circumference.
	• The ultimate purpose of studying the pathophysiological factors to make an accurate nutritional diagnosis and to perform a medical and nutritional intervention aimed at preventing or treating malnutrition associated with CCLD is to improve the growth, development, and quality of life of affected children.

*CCLD, chronic cholestatic liver disease*.

## Author Contributions

AL-H planned the nutritional issues to be discussed in this review and drafted the manuscript. EC-S carried out the scoping review and contributed to the writing of the document. Both authors contributed to the article and approved the submitted version.

## Conflict of Interest

The authors declare that the research was conducted in the absence of any commercial or financial relationships that could be construed as a potential conflict of interest.

## Publisher's Note

All claims expressed in this article are solely those of the authors and do not necessarily represent those of their affiliated organizations, or those of the publisher, the editors and the reviewers. Any product that may be evaluated in this article, or claim that may be made by its manufacturer, is not guaranteed or endorsed by the publisher.
